# Drug related problems in the neonatal intensive care unit: incidence, characterization and clinical relevance

**DOI:** 10.1186/s12887-019-1499-2

**Published:** 2019-04-26

**Authors:** Ramon Duarte Leopoldino, Marco Tavares Santos, Tatiana Xavier Costa, Rand Randall Martins, António Gouveia Oliveira

**Affiliations:** 1 Department of Pharmacy, Universidade Federal do Rio Grande do Norte, Av. General Gustavo Cordeiro de Farias, s/n. Petrópolis, Natal, RN 59012-570 Brazil; 2 Maternity School Januário Cicco, Universidade Federal do Rio Grande do Norte, Av. Nilo Peçanha, 259. Petrópolis, Natal, RN 59012-310 Brazil

**Keywords:** Adverse drug events, Critical care, Drug therapy, Medication errors, Neonate

## Abstract

**Background:**

Any event involving drug therapy that may interfere in a patient’s desired clinical outcome is called a drug related problem (DRP). DRP are very common in intensive therapy, however, little is known about DRP in the Neonatal Intensive Care Unit (NICU). The purpose of this study was to determine the incidence of DRPs in NICU patients and to characterize DRPs according to type, cause and corresponding pharmaceutical conducts.

**Methods:**

Prospective observational study conducted in the NICU at a teaching hospital in Brazil from January 2014 to November 2016. The data were collected from the records of the clinical pharmacy service, excluding neonates admitted for less than 24 h and those who had no drugs prescribed. DRPs were classified according to the Pharmaceutical Care Network Europe system and evaluated for relevance-safety.

**Results:**

Six hundred neonates were included in the study, with mean gestational age of 31.9 ± 4.1 weeks and mean birth weight of 1779 ± 885 g. The incidence of DRPs in the NICU was 6.8% patient-days (95%CI 6.2–7.3%) and affected 59.8% of neonates (95% CI 55.8–63.8%). Sub-optimal effect (52.8%) and inappropriate dose selection (39.75%) were the most common problem and cause, respectively. Anti-infectives was the medication class most involved in DRPs. More than one-third of neonates were exposed to DRP of significant or high safety-relevance. Most of the pharmaceutical interventions were related with drug prescription, with over 90% acceptance by attending physicians.

**Conclusion:**

DRP are common in NICU, predominating problems of sub-optimal treatment, mainly due to inappropriate dose selection.

**Electronic supplementary material:**

The online version of this article (10.1186/s12887-019-1499-2) contains supplementary material, which is available to authorized users.

## Background

Drug therapy may be implicated in undesirable effects and potential injury to patient health, even though benefits are expected. Such eventualities, as well as any other circumstances that interfere with the drug therapy of patients, are called drug related problems (DRP) [1]. In general lines, DRPs may involve errors in the drug therapy process (medication errors) or may result from a harmful effect of the drug (adverse drug reaction) [2].

When DRPs are not identified, and therefore not resolved, they can aggravate the patient’s clinical condition, extend the length of stay and, in extreme cases, lead to a fatal outcome. Consequently, DRPs often lead to an increase in healthcare costs [3, 4]. In 2013, Tasaka et al. [5] predicted a cost of more than 30 million dollars with DRPs in Japanese hospitals. Therefore, knowing the risks to which patients are exposed is of great importance to achieve safer drug therapy and, consequently, better disease management.

DRPs are very common in adult intensive care [6], as expected because of the seriousness of the patient’s health condition and the complexity of drug therapy. In Pediatrics there is not much information, but it has been estimated that from 20 to 50% of children suffer some DRP during the hospital stay [7–10], although the majority of DRP are preventable [7, 8]. In neonates admitted to Neonatal Intensive Care Units (NICU) the lack of information is even more critical. To the best of our knowledge, there are no published studies on DRP focusing specifically on neonates, although it is believed that DRPs are more frequent and severe in neonates than in older children and adults [11–13]. This is because of the physiological immaturity, which interferes with drug pharmacokinetics (absorption, distribution, metabolism and excretion), the rapid body growth combined with the administration of drug doses based on body weight, and the frequent use of off-label drugs [14, 15]. The latter condition is even more worrying due to the lack of studies adequately addressing the therapeutic needs of neonates [16].

Therefore, the main objectives of our study were to determine the incidence of DRPs in neonates during their stay in a NICU and to characterize DRPs by type, cause and corresponding pharmaceutical conduct. The secondary objectives were to identify the class of medicines most involved with DRPs, to assess the clinical relevance of DRPs and to measure the acceptability of pharmaceutical interventions.

## Methods

From January 2014 to November 2016 we prospectively conducted an observational, cohort study in the NICU of a teaching maternity hospital that is a referral centre for high-risk pregnancy. During the study period, all the newborns who were admitted to the NICU for a stay longer than 24 h and who were prescribed with at least one medicine were included in the study. Electrolyte and parenteral nutrition solutions, whole blood or blood products, oxygen therapy and diagnostic agents were not considered as medicines. Vitamin and mineral supplements were also not considered, except for ferrous sulfate and phytonadione because these supplements have a well-defined dosage and therapeutic indication, and require pharmacotherapeutic follow-up.

The following data were collected from the records of all neonates included in the study: sex, gestational age, birth weight and length of NICU stay. In each patient, the number of prescribed drugs and the occurrence of DRPs were recorded daily throughout the NICU stay.

The NICU clinical pharmacy team, consisting of a chief pharmacist and four pharmacy residents, assessed patients daily for the occurrence of potential DRPs, through the review of medical charts, physician orders and nursing reports. The information on DRPs was recorded in pharmacotherapy follow-up sheets, which were reviewed independently by two clinical pharmacists. Only DRPs consistent with the Pharmaceutical Care Network Europe (PCNE) definition of DRP (“event or circumstance involving drug therapy that actually or potentially interferes with desired health outcome” [1]) were considered for the study. Inquiries from attending physicians or other healthcare professionals about pharmacotherapy were not considered as DRPs. Adverse events for which there were conclusive reports in the literature relating them to one of the drugs being administered were considered adverse drug reactions. The DRPs were also independently classified by the two evaluators, according to the PCNE version 6.2 system (Additional file 1), by problems, causes and pharmaceutical interventions [1]. In case of disagreement between the evaluators, a third pharmacist was consulted. The pharmaceutical interventions were evaluated for acceptance by other health professionals (for details of the process of identification, validation and classification of DRPs, see Additional file 2).

In order to evaluate the clinical significance of DRPs in neonates, each DRP was classified according to its safety-relevance, that is, the potential risk of a DRP for causing serious damage to the patient’s health, by three clinical pharmacists based on the tool developed by Lewinski et al. [17]. That tool combines an assessment of the most serious damage that a DRP may cause, on one hand, and of the probability of that damage, on the other hand. To apply that tool, the potential injuries caused by each DRP were identified through consultation of the 2011 Neofax® textbook (Thomson Reuters, New York, USA) and the Micromedex® (Truven Health Analytics, Michigan, USA) and Uptodate® (WoltersKluwer, AlphenaandenRijn, NL) databases. Each potential injury was classified according to its degree of severity as mild, significant and serious/irreversible, and only the most severe injury was considered. Then, the probability of damage of that injury was estimated, based on the clinical experience of the evaluators, and categorized as low (< 2%), medium (2–20%) and high (20–100%). The safety-relevance score of each DRP, classified as minor, significant or high, is obtained with that tool, which is actually a matrix combining the degree of severity and the probability of damage. For example, a DRP has minor safety-relevance when it may cause minor damage or when it has low probability of causing significant damage. A DRP has significant safety-relevance when it has medium or high probability of causing significant damage or when this has low probability of causing serious damage. A DRP has high safety-relevance when it has medium or high probability of causing serious damage. If the severity of the damage is zero or the probability is zero for all listed damages, the DRP has no clinical relevance.

### Statistical analysis

As no information existed on the proportion of newborns experiencing a DRP during hospitalization in a NICU, the worst scenario, in terms of required sample size, of a 50% proportion was assumed. A sample size of 600 neonates would provide estimates with a maximum error of ±4% points with 95% confidence. Interval variables are described by mean ± standard deviation, binary variables by absolute and relative frequency, and time variables by median and range. The incidence density of DRP was expressed per 100 patient-days with Poisson 95% confidence interval (CI). Statistical analysis was performed with Stata 11 (Stata Corporation, College Station, TX, USA).

## Results

During the study period, a total of 634 newborns were admitted to the NICU. Of these, 19 newborns were not eligible for the study because they had no medication prescribed (17 patients) or because the length of stay was less than 24 h (2 patients). From the 615 newborns included in the study, 15 newborns (2.4%) were excluded from the analysis because they had missing pharmacotherapy data. Therefore, 600 newborns were retained for analysis. The study population consisted of 313 males (52.2%) with a mean gestational age of 31.9 ± 4.1 weeks and a mean birth weight of 1779 ± 885 g. Newborns remained hospitalized in the NICU for a median of 14 days (range 1–278 days). The in-NICU mortality rate was 12.7% (76 deaths). A summary of demographic and clinical characteristics is shown in Table 1.

**Table 1:** Demographic and clinical characteristics of the study population

Characteristics	Value	
Gestational age in weeks (m, sd)	31.9	4.1
Male gender (n, %)	313	52.2
Birth weight in grams (m, sd)	1779	885
Length of NICU stay in days (median, range)	14	1–278
Death (n, %)	76	12.7

*m* mean, *sd* standard deviation

A total of 1142 DRPs were identified in the study. DRPs affected 359/600 newborns (59.8, 95% CI 55.8–63.8%) with a mean of 1.9 ± 2.6 DRPs per patient. The NICU incidence density of DRPs was 6.8% patient-days (95% CI 6.2–7.3%). Treatment ineffectiveness (619, 54.2%) and adverse reaction (472, 41.4%) were the most frequent DRPs (Table 2). The main cause of DRPs, classified according to PCNE 6.2, was C3 – inappropriate dose selection (454, 39.75%), mostly due to C3.1 – drug dose too low (154, 13.5%) and C3.3 – dosage regimen not frequent enough (124, 10.9%). Another frequent cause was C5 – inappropriate drug use process (373, 32.7%), especially inappropriate C5.5 – wrong drug administered (164, 14.4%). Table 3 gives more details of the causes of DRP.

**Table 2:** Profile of drug related problems (DRP) according to the PCNE classification version 6.2

DRP profile (*n* = 1142)	Value	
Patients with DRP (n, %)	359	59.8
DRPs per patient (m, sd)	1.9	2.6
DRP incidence (% patient-days, 95%CI)	6.8	6.2–7.3
Distribution of DRP by Problems (n, %)		
P1 – Treatment effectiveness	619	54.2
P1.2 – Effect of drug treatment not optimal	603	52.8
P1.4 – Untreated indication	15	1.3
P1.1 –No effect of drug treatment/ therapy failure	1	0.1
P2 – Adverse reactions	472	41.4
P2.3 – Toxic adverse drug-event	267	23.4
P2.1 – Non-allergic adverse drug event	204	17.9
P2.2 – Allergic adverse drug event	1	0.1
P3 – Treatment costs	10	0.9
P3.2 – Unnecessary drug-treatment	10	0.9
P4 – Others	41	3.6
P4.2 – Unclear problem/complaint	41	3.6

*m* mean, *sd* standard deviation, *CI* confidence interval

**Table 3:** Main causes of drug related problems (DRP) according to the PCNE classification version 6.2

Causes of DRP (*n* = 1142)	n	%
C3- Dose selection	454	39.7
C3.1- Drug dose too low	154	13.5
C3.3- Dosage regimen not frequent enough	124	10.9
C3.2- Drug dose too high	117	10.2
C3.7- Deterioration or improvement of disease state requiring dose adjustment	38	3.3
C3.4- Dosage regimen too frequent	21	1.8
C5- Drug use process	373	32.7
C5.5- Wrong drug administered	164	14.4
C5.1- Inappropriate timing of administration and/or dosing intervals	103	9.0
C5.4- Drug not administered at all	70	6.1
C5.2- Drug under-administered	36	3.2
C6- Logistics	187	16.4
C6.2- Prescription error (necessary information missing)	157	13.8
C6.1- Prescribed drug not available	30	2.6
C1- Drug selection	52	4.5
C1.3- Inappropriate combination of drugs, or drugs and food	35	3.1
C1.8- Synergistic or preventive drug required and not given	6	0.5
C1.4- Inappropriate duplication of therapeutic group or active ingredient	4	0.3
C1.5- Indication for drug treatment not noticed	4	0.3
C1.2- No indication for drug	2	0.2
C1.1- Inappropriate drug	1	0.1
C8- Other	76	6.7
C8.1- Others specific causes	76	6.7
C8.2- No obvious cause	0	0

Newborns were prescribed with 4970 medicines, with an average of 8.28 ± 6.11 medicines per patient, of which 1273 were involved in the occurrence of DRPs. The drug classes most involved in DRPs were anti-infectives (729, 57.3%), cardiovascular agents (202, 15.9%) and respiratory agents (131, 10.3%) and these accounted for over 80% of DRP in the NICU. Specifically, gentamicin (220, 17.3%), aminophyline (104, 8.2%) e meropenem (101, 7.9%) were the medicines most involved in DRP. The ten medicines most involved in DRP are shown in Table 4.

**Table 4:** The ten medicines most involved in drug related problems (DRP) distributed by causes of DRP

Medicines	Cases of DRP (*n* = 1273)	Causes of DRP			
		Dose selection	Drug selection	Drug use	Logistics
Gentamicin	220 (17.3%)	137 (10.76%)		78 (6.13%)	3 (0.24%)
Aminophylline	104 (8.2%)	24 (1.89%)	2 (0.16%)	59 (4.63%)	9 (0.71%)
Meropenem	101 (7.9%)	40 (3.14%)	1 (0.08%)	25 (1.96%)	28 (2.20%)
Vancomycin	99 (7.8%)	31 (2.44%)	1 (0.08%)	24 (1.89%)	40 (3.14%)
Amikacin	75 (5.9%)	47 (3.69%)		16 (1.26%)	8 (0.63%)
Dobutamine	58 (4.6%)	2 (0.16%)		51 (4.01%)	2 (0.16%)
Ampicillin	47 (3.7%)	27 (2.12%)		12 (0.94%)	8 (0.63%)
Furosemide	46 (3.6%)	9 (0.71%)	10 (0.79%)	24 (1.89%)	3 (0.24%)
Amphotericin B	44 (3.5%)	6 (0.47%)		20 (1.57%)	10 (0.79%)
Cefepime	42 (3.3%)	16 (1.26%)		11 (0.86%)	14 (1.10%)

Most DRPs resulted in an intervention on patient’s pharmacotherapy (960, 86.1%). Of these interventions, 641 were advice to the physicians and 319 were advice to the nurses. The proportion of interventions accepted was, respectively, 90.8% (582/641) and 97.8% (312/319), with an overall acceptance rate of 93.1% (894/960).

Table 5 presents the safety-relevance of the DRPs. From 1142 DRPs, 386 (33.8%) had significant and 40 (3.5%) had high safety-relevance. Three hundred and four (50.7, 95% CI 46.6%; 54.7%) neonates were exposed to 642 (56.2%) DRPs of minor safety-relevance, most often potential ineffectiveness of ferrous sulfate for the treatment of mild anemia. DRPs of high or significant safety-relevance affected 206 (34.3, 95% CI 30.5–38.3%) neonates, the most common being potential toxicity of vancomycin due to non-dose adjustment according to renal impairment (high relevance) and the potential ineffectiveness of gentamicin due to administration of doses lower than adequate for the treatment of sepsis (significant relevance). Only 74 (6.5%) DRPs in 61 (10.2, 95% CI 7.9–12.9%) neonates had no relevance, with waste in the preparation (reconstitution and dilution) of amphotericin B (problem – P3.1) being the most common DRP.

**Table 5:** Safety-relevance of drug related problems (DRP)

Safety-relevance of DRP	Cases of DRP (*n* = 1142)		Patients exposed (*n* = 600)	
	n	%	n	%
Minor	642	56.2	304	50.7
Significant	386	33.8	196	32.7
High	40	3.5	31	5.2
None	74	6.5	61	10.2

## Discussion

Among the main findings of the study is the estimate of the incidence of DRPs in NICU of 6.8 per 100 patient-days, a result not previously described in the literature. The main cause of DRPs involved the prescription of inappropriate doses, which translated into potential problems of therapeutic effectiveness and drug toxicity. Anti-infectives, especially gentamicin, were the drugs most involved in DRPs. Another important fact was the significant occurrence of DRPs with clinical relevance, with more than one third of newborns at great risk of significant damage. Also noteworthy was the high acceptability of interventions proposed by the pharmacist to the NICU physicians and nurses. Several methodology features give strength to our results, namely the cohort design, the prospective data collection, the large number of patients and the adoption of a standard DRP classification system. The PCNE classification system was chosen because of its clear hierarchical structure of problems and causes, as well as of its wide application in DRP research studies [18].

A very small number of studies have evaluated DRP in neonates, and none has been specifically designed for this population. In general pediatrics, we have found only three studies evaluating the frequency and nature of DRPs in hospitalized children. Two prospective cohort studies involving less than 120 neonates, one in Hong Kong and another in the United Kingdom and Saudi Arabia, found an overall prevalence of DRP of less than 50% [7, 8]. Both studies had a duration of 3 months and adopted the PCNE definition of DRPs. In these studies, DRPs were identified by review of medical charts and physician orders, but DRPs occurring during weekends were not considered. With the same DRP identification method, but using another classification of DRPs, Birarra et al. [10] found an overall prevalence of 30% in pediatric wards at a hospital in Ethiopia. The study was a cross-sectional study involving 285 children, but only 21 neonates, for 3 months. Although our work has adopted a method similar to the above studies, our prevalence was higher, with almost 60% of patients experiencing at least one DRP. Importantly, our study had a greater number of neonates as well as a longer recruitment period (3 years).

It should be noticed that in our study the DRPs occurred even though the NICU has an institutional clinical practice guideline that includes dosing guidelines for all drugs. Thus, one of the main reasons for the high occurrence of DRPs in NICUs is the physiological immaturity of neonates. Neonates have characteristics that change the pharmacokinetics of many drugs, with a significant impact on the pharmacotherapy. This population, unlike adults, has a low plasma protein concentration and a higher percentage of body water, in addition to decreased liver metabolism and renal clearance [19]. These characteristics vary constantly along the growth and maturation of the neonate making it difficult to establish the adequate dose for each case. Consequently, the risk of either drug ineffectiveness or toxicity is always present in neonates [15, 20]. Another aspect of neonatal drug therapy is that medicinal formulations appropriate for this population are rare and, therefore, the dilution of medications for adult use is a common and necessary practice, a process that may also lead to subdoses as well as overdoses [21].

Accordingly, several studies in the pediatric population [7–10], as well as our study, have shown a predominance of DRPs with the potential for therapeutic ineffectiveness, mainly due to inappropriate dose selection. The main medicine that illustrates the difficulty in establishing optimal dosage schedules is gentamicin. This medicine is preferably distributed in aqueous compartments and is excreted unchanged almost exclusively by the kidneys [19, 20]. Because of these characteristics, neonates tend to have lower serum concentrations due to the progressive renal maturation and to the large body water volume during the first days of life. Hence, it is recommended that gentamicin dose be adjusted frequently as a function of postnatal life [19]. In addition, the existence of multiple recommended dose regimens makes it difficult to prescribe gentamicin in neonatal practice [22, 23]. Such aspects explain why gentamicin was often involved in DRPs in our study, a finding consistent with other studies [8, 24–26].

Although less frequent than the problems of effectiveness, adverse reactions are also significant. In the first 72 h of life, the neonate may present a body weight reduction of more than 20% and the absence of dose adjustment of medicines contributes to a greater risk of toxicity [20]. However, most adverse reactions in our cohort were only potential, with only 22 actual adverse drug reactions affecting 4% of newborns.

In our study, we estimate that nearly nine out of ten DRPs were preventable. Most pharmaceutical interventions were related to drug prescription and almost all were accepted by the NICU team, a result similar to that reported in other papers [9, 27]. Before proposing an intervention, the pharmacist should always consider the condition of the patient as well as the resources offered by the hospital and the health professionals. Thus, for an intervention to be adequate, the severity of each DRP must be equated. A study has shown that, compared to adults, pediatric patients are at a higher risk of severe DRPs, but published information on the actual risk of DRPs is limited [12]. In our study, the safety-relevance analysis of DRPs showed that more than one third of neonates are exposed to DRPs with a considerable risk of causing moderate to severe injury, representing almost 40% of all identified DRPs. Using a different tool, Ibrahim et al. [9] and Rashed et al. [7, 8] observed that 30 to 50% of the DRPs were of moderate severity, but no severe DRPs were identified.

In addition to the performance of clinical pharmacists in the NICU, there are other strategies to reduce DRP as computerized physician order entry integrated with clinical decision support, barcode dispensing and administration system, reporting system of errors and adverse events and programs of training and continuing education [28]. All those tools are in use at our NICU, except that the computerized physician order entry does not yet automatically check doses.

This study has some limitations. Firstly, the study was conducted in a single NICU, which may limit generalization of the results. However, the large majority of published studies on this and related topics were also single center studies. Secondly, the data were collected from secondary sources, including pharmacotherapy records, clinical charts, nursing records, physician orders and pharmacovigilance notifications, which might decrease data quality, but this was likely to have little impact on the study results because patient data was examined and evaluated prospectively and as was being recorded. Thirdly, the evaluation of the safety-relevance of the DRPs was made only by pharmacists with an unvalidated tool and supported by Neofax® 2011, which in part may have compromised this analysis. However, in the context of DRPs, we consider the relevance-safety analysis more adequate than just severity, because it combines the severity of a potential adverse event with its likelihood, offering a better measure of the potential risk to which the patient was exposed. Lastly, the therapeutic drug monitoring service is not a usual practice in our NICU, so it is possible that some DRPs have been underestimated.

Future research in this topic should preferably make efforts to include the evaluation of clinical outcomes related to DRP and to analyze the actual risk of DRP instead of the potential risk, as was the case in this study and in several other published studies. Studies on risk factors for DRP are also needed.

## **Conclu**s**ion**

In conclusion, we observed that DRPs are common in the NICU, predominating potential problems of drug therapy effectiveness, mainly due to inappropriate dose selection. The most problematic drugs are the anti-infectives, notably gentamicin, with an important proportion of DRPs of significant or high clinical relevance. Pharmaceutical interventions near the healthcare team are well accepted.

## Additional files

Additional file 1: PCNE systems v6.2 and operational definitions of the study for the classification of drug related problems (DRP). (DOCX 25 kb)

Additional file 2: Process of identification, validation and classification of drug related problems (DRP). (DOCX 17 kb)

## Abbreviations

CI: Confidence interval
DRP: Drug related problems
NICU: Neonatal Intensive Care Unit
PCNE: Pharmaceutical Care Europe
