# Supplementary material for: Drug related problems in the neonatal intensive care unit: incidence, characterization and clinical relevance
Source: BMC Pediatr. 2019 Apr 26;19:134. doi: 10.1186/s12887-019-1499-2 (PMC6485091; doi:10.1186/s12887-019-1499-2)
Supplement: Supplementary file 1 — PCNE systems v6.2 and operational definitions of the study for the classification of drug related problems (DRP). (DOCX 25 kb) [file 12887_2019_1499_MOESM1_ESM.docx]

Additional file 1 – PCNE systems v6.2 and operational definitions of the study for the classification of drug related problems (DRP)

| **Code – Categories** | **Operational definition** |
| --- | --- |
| Problems | |
| P1 – Treatment effectiveness | There is a (potential) problem with the (lack of) effect of the pharmacotherapy. |
| P1.1 – No effect of drug treatment/therapy failure | The drug treatment cannot lead or does not lead to the improvement of the patient's symptoms (e.g. early sepsis not responsive to treatment of ampicillin and gentamicin). |
| P1.2 – Effect of drug treatment not optimal | The drug treatment may lead or lead to a partial improvement of the patient's symptoms (e.g. paracetamol sub-dose leading to a partial pain relief). |
| P1.3 – Wrong effect of drug treatment | Not applicable for the study. |
| P1.4 – Untreated indication | There are symptoms that need treatment but are not being treated at the moment (e.g. patient has a fever but is not in drug treatment). |
| P2 – Adverse reactions | Patient suffers, or will possibly suffer, from an adverse drug event. |
| P2.1 – Non-allergic adverse drug event | The drug treatment may be related to an unintended, non-allergic adverse event with doses normally used for the intended indication (e.g. tachycardia reportedly related to the use of caffeine). |
| P2.2 – Allergic adverse drug event | The drug treatment may be related to an unintended, allergic adverse event with doses normally used for the intended indication (e.g. skin rash reportedly related to the use of penicillin). |
| P2.3 – Toxic adverse drug-event | The drug treatment may lead or lead to an unintended adverse event occurring in doses higher than that normally used for the intended indication (e.g. captopril overdose leading to hypotension). |
| P3 – Treatment costs | The drug treatment is more expensive than necessary. |
| P3.1 – Drug treatment more costly than necessary | The medicine is more expensive than other medicines available or there is a waste in its preparation (reconstitution and dilution). |
| P3.2 – Unnecessary drug-treatment | The prescribed medication is not necessary or no longer necessary. |
| P4 – Others | Other problems not specified above |
| P4.1 – Patient dissatisfied with therapy despite optimal clinical and economic treatment outcomes | - |
| P4.2 – Unclear problem/complaint | Problem not clarified or without defined classification. |
| Causes | |
| C1 – Drug selection | The cause of the DRP is related to the selection of the drug. |
| C1.1 – Inappropriate drug (including contraindication) | Selected drug is inappropriate for the intended indication or is contraindicated for the patient (e.g. ampicillin prescribed to allergic patient). |
| C1.2 – No indication for drug | There is no indication for the selected drug. |
| C1.3 – Inappropriate combination of drugs, or drugs and food | The selected drug interacts or may interact physically, physico-chemical or chemically with other drugs or foods (e.g. patient is receiving ciprofloxacin and fluconazole, medicines that may increase the risk of QT interval prolongation and, consequently, ventricular arrhythmias). |
| C1.4 – Inappropriate duplication of therapeutic group or active ingredient | The physician order inappropriately has medicines of same therapeutic group or active ingredients to treat different symptoms (e.g. ibuprofen indicated for closure of the ductus arteriosus and paracetamol indicated for fever present in the same physician order). |
| C1.5 – Indication for drug-treatment not noticed | The appropriate drug is not used to treat the symptom because the existence of the symptom is not noticed (e.g. patient has a fever that is not noticed and, therefore, is not in drug treatment). |
| C1.6 – Too many drugs prescribed for indication | The physician order inappropriately has medicines indicated to treat the same symptoms (e.g. ranitidine and omeprazole both indicated for gastrointestinal haemorrhage present in the same physician order). |
| C1.7 – More cost-effective drug available | There are cheaper and effective (or more effective) medications to treat the symptoms. |
| C1.8 – Synergistic/preventive drug required and not given | There is a requirement to use a medication to improve an existing treatment or to prevent the development of another symptom, but it is not used. (e.g. ferrous sulfate requirement for the prevention of anemia). |
| C1.8 – New indication for drug treatment presented | The patient presents a new symptom that is not being treated (e.g. patient has a recent fever and requires drug treatment). |
| C2 – Drug form | The cause of the DRP is related to the selection of the drug form. |
| C2.1 – Inappropriate drug form | The drug has an inappropriate form and/or formula for the patient (e.g. oral caffeine solution prescribed for neonate with feeding intolerance). |
| C3 – Dose selection | The cause of the DRP is related to the selection of the dosage schedule. |
| C3.1 – Drug dose too low | Selected dose is 20% lower than the minimum dose defined for the intended indication (e.g. cefepime prescribed 45 mg/kg instead of 60 mg/kg daily). |
| C3.2 – Drug dose too high | Selected dose is 20% higher than the maximum dose defined for the intended indication (e.g. oxacillin prescribed 150 mg/kg instead of 100 mg/kg daily). |
| C3.4 – Dosage regimen not frequent enough | Selected dosing frequency is less than that defined for the intended indication (e.g. gentamicin prescribed every 48 hours instead of every 36 hours). |
| C3.5 – Dosage regimen too frequent | Selected dosing frequency is higher than that defined for the intended indication (e.g. amikacin prescribed every 24 hours instead of every 36 hours). |
| C3.6 – No therapeutic drug monitoring | Monitoring serum levels of the drug is required, but it is not done. |
| C3.7 – Pharmacokinetic problem requiring dose adjustment | Not applicable for the study. |
| C3.8 – Deterioration/improvement of disease state requiring dose adjustment | Change in disease state requiring dose adjustment (e.g. vancomycin dose adjustment because of the improvement in renal function in patients with renal impairment). |
| C4 – Treatment duration | The cause of the DRP is related to the duration of therapy. |
| C4.1 – Duration of treatment too short | Duration of treatment is shorter than that defined for the indication treated (e.g. penicillin prescribed for eight days instead of ten days). |
| C4.2 – Duration of treatment too long | Duration of treatment is longer than that defined for the indication treated (e.g. meropenem prescribed for sixteen days instead of fourteen days). |
| C5 – Drug use process | The cause of the DRP can be related to the way the patient uses the drug, in spite of proper dosage instructions (on the label). |
| C5.1 – Inappropriate timing of administration and/or dosing intervals | Drug administered at wrong times or intervals (e.g. gentamicin dose scheduled for 16 hours but administered at 18 hours). |
| C5.2 – Drug under-administered | Drug administered at a frequency lower than the physician order (e.g. ranitidine prescribed twice daily but administered only once). |
| C5.3 – Drug over-administered | Drug administered at a frequency higher than the physician order (e.g. aminophylline prescribed twice daily but administered three time). |
| C5.4 – Drug not taken/administered at all | Drug dose is not administered in full (e.g. ampicillin dose administered in half). |
| C5.5 – Wrong drug administered | Drug is administered wrong (e.g. norepinephrine was administered in the wrong route). |
| C5.6 – Drug abused (unregulated overuse) | Not applicable for the study. |
| C5.7 – Patient unable to use drug/form as directed | Not applicable for the study. |
| C6 – Logistics | The cause of the DRP can be related to the logistics of the prescribing and dispensing process. |
| C6.1 – Prescribed drug not available | Prescribed drug is not available in the institution and there is no other effective drug (e.g. ursodiol is prescribed but not available in the hospital and there is no other drug alternative). |
| C6.2 – Prescribing error (necessary information missing) | Missing necessary information on the drug prescription that may generate a medication error (e.g. vancomycin is prescribed, but there is no information on the minimum recommended time for administration). |
| C6.3 – Dispensing error (wrong drug or dose dispensed) | Drug is dispensed wrong or dispensed in the wrong dosage form or dose (e.g. dispensed intravenous furosemide instead of oral). |
| C7 – Patient | The cause of the DRP can be related to the personality or behaviour of the patient. |
| C7.1 – Patient forgets to use/take drug | Not applicable for the study. |
| C7.2 – Patient uses unnecessary drug | Not applicable for the study. |
| C7.3 – Patient takes food that interacts | Not applicable for the study. |
| C7.4 – Patient stored drug inappropriately | Not applicable for the study. |
| C8 – Others | Other causes not specified above. |
| C8.1 – Others specific causes | The problem arises due to other specific causes (e.g. adverse events related to alprostadil, cefepime prepared wrongly and etc.). |
| C8.2 – No obvious cause | Not applicable for the study. |
