# Supplementary material for: Drug related problems in the neonatal intensive care unit: incidence, characterization and clinical relevance
Source: BMC Pediatr. 2019 Apr 26;19:134. doi: 10.1186/s12887-019-1499-2 (PMC6485091; doi:10.1186/s12887-019-1499-2)
Supplement: Supplementary file 2 — Process of identification, validation and classification of drug related problems (DRP). (DOCX 17 kb) [file 12887_2019_1499_MOESM2_ESM.docx]

Additional file 2 – Process of identification, validation and classification of drug related problems (DRP)

| **Professionals responsible** | **Stage** | **Process** |
| --- | --- | --- |
| One chief pharmacist and four pharmacy residents | 1 | Review of medical charts, physician orders and nursing reports based on the institutional clinical practice guideline. |
|  | 2 | Identification of DRPs and elaboration of interventions.* |
|  | 3 | Record of DRP and interventions in pharmacotherapy follow-up sheets. |
| Two independent pharmacists and a third pharmacist in case of disagreement. | 4 | Validation of DRPs: assessment of DRPs regarding compliance with the PCNE definition.** |
|  | 5 | Classification of DRPs according to the PCNE version 6.2 system. |

* Pharmaceutical interventions related to prescriptions drugs and adverse events were directed to the physicians and those related to the drug preparation and administration were directed to the nurse. Systematic failures were passed on to all NICU staff.

** DRP is defined according to Pharmaceutical Care Network Europe (PCNE) as “event or circumstance involving drug therapy that actually or potentially interferes with desired health outcome” [1].
